# Per- and polyfluoroalkyl substances in commercially important marine species of fish and shellfish from Alaska and implications for human exposure

**DOI:** 10.1016/j.toxrep.2026.102302

**Published:** 2026-06-25

**Authors:** Christoff G. Furin, John Burrows, Andrew P. Cyr, Sarah Coburn, Robert F. Gerlach

**Affiliations:** a Alaska Department of Environmental Conservation, Division of Environmental Health, Office of the State Veterinarian, 5251 Dr. Martin Luther King Jr. Ave., Anchorage, AK 99507, USA; b Alaska Seafood Marketing Institute, 311 N. Franklin St. Suite 200, Juneau, AK 99801, USA; c Alaska Department of Health, Division of Public Health, Section of Epidemiology, 3601 C Street Suite 540, Anchorage, AK 99503, USA

**Keywords:** PFAS, Risk assessment, Food chain, Salmon, Seafood consumption, Risk-Benefit analysis

## Abstract

Per- and polyfluoroalkyl substances (PFAS) are persistent environmental synthetic contaminants associated with some adverse health effects under certain exposure conditions. Dietary sources, particularly seafood, have been identified as a potentially significant contributor to PFAS body burden. Muscle tissue from several species of fish and shellfish harvested in Alaska were analyzed for 40 PFAS analytes. Ten analytes were detected above the method detection limit (MDL) with PFTrDA and PFUnA being the most frequently detected. Concentrations of all analytes ranged from below the detection limit to 0.809 ng/g wet weight with most being below the limit of quantification (LOQ). Overall, PFAS concentrations were low or non-detect, consistent with other market basket studies of seafood. Although no national PFAS-specific fish consumption thresholds are currently established, ongoing surveillance of PFAS in commercially and recreationally harvested seafood provides critical exposure data to inform risk assessment and regulatory decision-making.

## Highlights

• Several species of fish and shellfish commonly caught in Alaska’s waters were tested for PFAS
• PFAS were either below the MDL or at low levels when found. Only ten of the 40 compounds were detected.
• Fish caught in Alaskan marine waters are not a significant source of PFAS

## 1 Introduction

Per- and polyfluoroalkyl substances (PFAS) are nearly ubiquitous persistent environmental contaminants that have been used in many industrial processes and consumer products beginning in the 1940s. PFAS chemical properties make them useful as surfactants, with applications for water and oil resistance, fire retardants, high-temperature-resistant coatings, and friction reduction. They are found in many consumer products including non-stick cookware, stain resistant treatments, waterproof clothing coatings, food packaging, and many other products and applications [39]. PFAS are anthropogenic organic compounds containing carbon and fluorine [12], [11]. Carbon-fluorine bonds are known to be chemically stable which contributes to the persistence of PFAS compounds in the environment. Long-chain (C6-C14) PFAS can bioaccumulate in organisms [13], and then biomagnify through the food web, resulting in higher concentrations in organisms at the top of the food chain. PFAS can also distribute differentially to various tissues, resulting in variable concentrations across tissues within an organism [10], [32], [9].

PFAS, particularly perfluorooctanesulfonic acid (PFOS) and perfluorooctanoic acid (PFOA), have been shown to be linked to some adverse health effects including reduced immunological response, hypothyroidism, altered liver enzyme and serum lipid profiles, and kidney disease [24], [63]. With the recognition of human health effects, a voluntary phase out of long-chain PFAS was initiated in 2000 by U.S. chemical manufacturers [39]. Alternative compounds have been introduced by the chemical industry to replace the phased-out compounds which include some short-chain (<6 C) PFAS such as perfluorobutanesulfonic acid (PFBS) and HFPO-DA (GenX). The health effects of these compounds are not well known and are an active area of research.

There are many routes of exposure to PFAS including dietary intake, inhalation of contaminated dust, use of personal care products (PCP), and drinking contaminated water [17], [19], [37], [65]. Due to the long half-lives in humans of some PFAS compounds, they tend to accumulate over time and can reach levels of potential health concern [53]. Indoor dust can be a significant exposure source, particularly for young children who are more likely to ingest dust incidentally [28], [34], [6]. Household materials such as carpets, upholstery, textiles, and stain repellants, can contribute PFAS to indoor dust [40]. Drinking water, if sourced near a contaminated site, can be a significant contributor to PFAS exposure [21], [5] and a significant portion of the U.S. population is likely exposed to some PFAS from drinking water sources [58]. Many contaminated drinking water sources, including the majority of Alaska’s contaminated sites [2], are a result of historical releases of aqueous film forming foam (AFFF). Both proximity to PFAS-contaminated sites and contaminated drinking water sources have been shown to increase exposure risk [17], [18], [19]. In the absence of a major contributing source, exposure likely comes from multiple routes.

Seafood has been described as a potential contributor to dietary PFAS exposure [19], [66]. PFAS levels in consumed fish and shellfish products are highly variable and depend on many factors including bioaccumulation via the environment through uptake from water or diet, and/or contamination post-harvest [25], [50]. Location, species, size/age, trophic level, and feeding habitat are some factors that contribute to environmental exposure of PFAS prior to harvest. Fish processing, food additives, cooking methods, and packaging occur after harvest, but they are factors that can contribute to PFAS exposure before reaching the consumer. Packaging is a known source of contamination to seafood for some PFAS compounds such as PFBS [49], [55], [8]. Evaluating exposure risk from seafood can aid in consumer choice and help in reducing contamination exposure risk.

Guidelines for fish consumption recommendations regarding PFAS exposure in the U.S. are complex and variable. The U.S. Environmental Protection Agency (USEPA) has recommended some PFAS compounds be monitored in freshwater fishes [69], but no federal fish consumption recommendations have been established for any PFAS compounds, individually or as a common chemical class. Some U.S. states with existing PFAS contaminated sites have set maximum tissue concentrations for fish consumption for select PFAS compounds (ex. MI, MN, WI, NJ). The European Union (EU) has established maximum levels for many fish species based on a tolerable weekly intake (TWI) of 4.4 ng/kg body weight per week for the sum of PFOS, PFOA, Perfluorononanoic acid (PFNA), and Perfluorohexanesulfonic acid (PFHxS) [20], [23]. The Food Safety Commission of Japan (FSCJ) established tolerable daily intakes (TDI) of 20 ng/kg body weight per day for PFOS and PFOA in 2025 [26]. Due to the potential health effects of PFAS, varying sources and geographical distribution, and potential exposure risk from some seafood, more relevant data are needed to establish consistent and likely patterns in exposure risk and provide sound consumption advice.

Fish and shellfish harvested in Alaska represent approximately 60% of seafood harvested in the United States [4]. Alaskan harvested fish are distributed around the globe, but the largest market is the United States, accounting for around 25% of seafood produced in Alaska [3]. Due to the large catch and global distribution, information about contaminant load and nutrient content is valuable for consumers when making choices and evaluating the risks versus benefits for seafood consumption. Species of fish and shellfish commercially harvested from Alaska’s marine waters include all five species of Pacific salmon (*Oncorhynchus* spp.), Pacific halibut (*Hippoglossus stenolepis*), Pacific cod (*Gadus macrocephalus*), sablefish (*Anoplopoma fimbria*), pollock (*Gadus chalcogrammus*), Northern rock sole (*Lepidopsetta polyxystra*), Alaska plaice (*Pleuronectes quadrituberculatus*), flathead sole (*Hippoglossoides elassodon*), yellowfin sole (*Limanda aspera*), rockfish (*Sebastes* spp.), king crab (*Paralithodes spp.*), bairdi and opilio crabs (*Chionoecetes spp.)*, scallops, shrimp, and more. Many of these same species are targeted by sport and subsistence harvesters from communities across the state.

Alaska does not have any industrial production of PFAS and the majority of contaminated sites in the state are the result of the discharge of AFFF [2]. Fish harvested in Alaska’s marine environment should have comparatively low body burdens of PFAS compounds based on the remoteness of fishing grounds and previous data from state surveillance studies which showed low or non-detectable PFAS levels in fish samples (Alaska Department of Environmental Conservation (ADEC), Supplementary tables). However, data quantifying PFAS levels in Alaskan fish are sparse. To better understand the current status and guide fish consumption recommendations, this study aimed to establish baseline concentration levels of PFAS compounds in Alaska caught species of fish and shellfish of commercial and subsistence importance. These species represent a large portion of the seafood caught, consumed, and exported from the state [4].

## 2 Methods

### 2.1 Sample design-

**Fig. 1:**
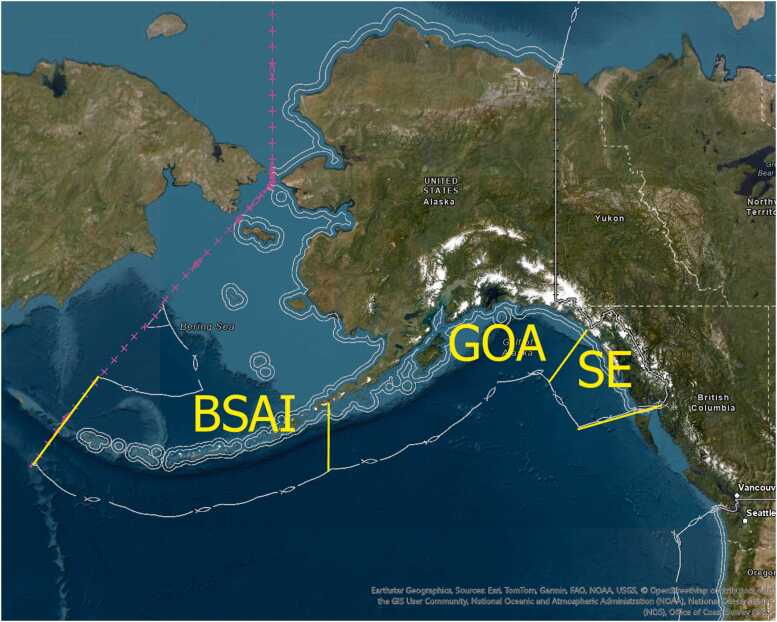
Map of Sampling areas, Bering Sea Aleutian Islands (BSAI), Gulf of Alaska (GOA) and Southeast Alaska (SE).

To obtain a representative sample for a species, the fish and shellfish were collected by commercial fishermen from three different regions of state waters commonly targeted by commercial fisheries. This included Southeast Alaska (SE), Gulf of Alaska (GOA) and the Bering Sea / Aleutian Islands (BSAI, Fig. 1). Species were selected based on their importance to commercial and subsistence fisheries in Alaska and availability from commercial seafood processors. Twenty fish of harvestable size from each of the three regions were sent to the State of Alaska Environmental Health Laboratory in Anchorage, AK for a total of 60 fish per species. Some species only occurred in certain regions therefore, more individuals were then collected to reach 60 when possible (see Table 1). Composite samples, created using 12 individuals per species that were randomly selected from all three regions, were then used for chemical analysis. This was done to model the supply of seafood to consumers and accounted for all fishing grounds where these species are commonly harvested.

**Table 1:** Species, samples numbers, and location information.

**Species**	**Bionomial Name**	**Count**	**Composites**	**Form**	**Regions**
Pacific Halibut	*Hippoglossus stenolepis*	60	5	H&G, Fillet	BSAI, GOA, SE
Pacific Cod	*Gadus macrocephalus*	60	5	Fillet	BSAI, SE
Pollock	*Gadus chalcogrammus*	84	7	Whole	BSAI, GOA
Sablefish	*Anoplopoma fimbria*	74	7	H&G	BSAI, GOA, SE
Pink Salmon	*Oncorhynchus gorbuscha*	60	5	Whole	BSAI, GOA, SE
Chum Salmon	*Oncorhynchus keta*	60	5	Whole, H&G	BSAI, GOA, SE
Coho Salmon	*Oncorhynchus kisutch*	60	5	Whole, H&G, Fillet	BSAI, GOA, SE
Chinook Salmon	*Oncorhynchus tshawytscha*	40	4	Whole, H&G	SE
Sockeye Salmon	*Oncorhynchus nerka*	60	5	Whole, H&G	BSAI, GOA, SE
Bairdi Crab	*Chionoecetes bairdi*	40	2	Cooked Sections	BSAI, SE
Red King Crab	*Paralithodes camtschaticus*	12	1	Cooked Sections	BSAI

H&G = Headed and gutted

BSAI = Bering Sea Aleutian Islands; GOA = Gulf of Alaska; SE = Southeast Alaska

### 2.2 Sample collection and processing-

Fish were provided directly from seafood processors operating in the three regions. The fish and shellfish samples were all caught in Alaskan waters. Sampling was done at the start of the supply chain and were minimally processed. When available, whole frozen fish or in-the-round fish were obtained. Some fish were dressed, i.e., head and/or internal organs removed. Frozen fillets were used when whole fish were not available (see Table 1). Filleting requires more handling and direct contact with packaging which can introduce contamination. All samples were taken directly from catch destined for sale and standard food safety practice for each species was used for handling and any processing. Samples were from the 2022–2024 seasons and shipped frozen in waxed cardboard boxes with a food grade plastic liner. Most were flash frozen and glazed to maintain quality. Crab were received as sections (legs and claws) that had been brine cooked and frozen.

Skinless fillets or muscle tissue was homogenized using a Robot Coupe Blixer© food processor (Ridgeland, MS, USA). All equipment in contact with the fish sample was decontaminated between samples using a cleaning protocol that consisted of laboratory grade detergent, 1% Nitric acid, hexane, methanol, and a final rinse with RO purified water. Composite samples were made and stored in 250 mL HDPE jars at −20 °C. Composites were prepared by combining equal aliquots of tissue from 12 individuals. Individual fish from each species were randomly selected from the pool of individuals, all of which were of harvestable size and representative of what would be available to the market. Samples were shipped in insulated shipping containers with dry ice overnight to SGS AXYS Analytical Services (Sidney, BC, Canada).

### 2.3 Analytical methods

Samples were analyzed using EPA method 1633 [68]. Briefly, samples of 2 g or less were pre-spiked with isotope-labeled surrogate standards (See Supplemental Table 1) and extracted with methanol and cleaned using solid phase extraction. Ultra-high-performance liquid chromatography/tandem mass spectrometry (LC-MS/MS) with reversed-phase C18 column using a solvent gradient was used to determine the quantity of PFAS in extracts from the homogenate samples. The column was coupled to a triple quadrupole mass spectrometer that ran at unit mass resolution in the Multiple Reaction Monitoring (MRM) mode, using negative electrospray ionization. Quantification was achieved by isotope-dilution-internal standard method using multipoint calibration curves. The reporting limit (RL), is synonymous with the limit of quantification (LOQ), was the lowest method calibration limit (LMCL), while the sample specific method detection limit (MDL) was determined by converting the area equivalent to 3.0 times the estimated chromatographic noise height to a concentration. If a result was below the LOQ, it was reported as an estimated value (J laboratory qualifier). PFAS compounds analyzed along with MDLs are listed in Supplemental Table 1. Quality assurance and quality control (QA/QC) included laboratory procedural blanks, matrix spikes, matrix spike duplicates, ongoing and precision sample, and lab duplicates. For more details about the laboratory analysis see Méndez et al. [46].

### 2.4 Data and statistics-

All statistics were performed using R version 4.5.1 [54]. When samples were non-detect (below the MDL) results were calculated by using half the MDL [38]. Means were calculated only when one or more composite samples for a species were above the LOQ (Table 3). When all samples for a species were less than the MDL, then it was considered non-detect. Results are reported as a wet weight and were not blank corrected but blank contamination, when present, is reported in Table 3.

## 3 Results

A total of eleven species were sampled, including nine species of finfish and two species of shellfish (Table 1). The average length and weight of fish received in whole form are shown in Table 2. Fifty-one composite samples were analyzed for PFAS and only ten individual compounds were detected in fish samples which included: N-Ethylperfluorooctanesulfonamidoethanol (N-EtFOSE), Perfluorodecanoic acid (PFDA), Perfluorododecanoic acid (PFDoA), PFHxS, PFNA, PFOA, PFOS, Perfluorooctanesulfonamide (PFOSA), Perfluorotridecanoic acid (PFTrDA), and Perfluoroundecanoic acid (PFUnA) (Table 3). A majority of results were below the LOQ. Laboratory blank contamination was also present in some QA/QC measures, but were below the method QC procedural blank level for all compounds in which there were blank detections; PFOS, PFOA, PFTrDA and PFUnA = ≤ 0.4 ng/sample, N-EtFOSE = ≤ 4.0 ng/sample (SGS-AXYS method MLA−110 Rev. 2 Ver. 16). The most commonly detected compounds in fish samples were PFUnA, PFTrDA and PFOS (56.9%, 41.2%, and 19.6% of samples, respectively; Table 3). The highest concentrations were in one pollock composite (PFOS), and two bairdi crab composites (PFTrDA and PFUnA) (Table 3 and Supplemental Figs 2 & 3). Bairdi crab had the most compounds detected (6), followed by pollock (5) and sockeye and chinook salmon (4 each). Of the 47 samples, 13 had no detectable PFASs and included all coho salmon, chum salmon, red king crab, and 3 of 5 pink salmon composites. No short-chain PFAS were detected.

**Table 2:** Species, Fork Length, and Body Weights (mean ± SD).

**Species**	n*	**Length (cm)**	**Weight (Kg)**
Pacific Halibut	5	83.7 ± 3.6	5.82 ± 0.1
Pacific Cod	0	NA	NA
Pollock	80	44.7 ± 5.2	0.82 ± 0.2
Sablefish	0	NA	#2.3 ± 0.8
Pink Salmon	54	52.8 ± 3.0	1.64 ± 0.3
Chum Salmon	40	64.2 ± 4.5	2.64 ± 0.6
Coho Salmon	12	69.8 ± 2.1	4.25 ± 0.6
Chinook Salmon	19	77.7 ± 3.3	7.54 ± 1.4
Sockeye Salmon	39	56.4 ± 4.7	1.05 ± 0.5
Bairdi Crab	0	NA	NA
Red King Crab	0	NA	NA

* Number of whole individuals received that could be measured

# Average weight of headed and gutted fish

**Table 3:** Percent detection, percent above the LOQ, and mean values (ng/g wet weight) of PFAS compounds in composite muscle samples of Alaska caught fish.

Sample	**n**	Statistic	NEtFOSE	PFDA	PFDoA	PFHxS	PFNA	PFOA	PFOS	PFOSA	PFTrDA	PFUnA
**C. bairdi Crab**	2	% Detect % > LOQ Mean±SD Min-max	0 0 ND NA	100 0 NA <LOQ	50 0 NA <LOQ	0 0 ND NA	100 0 NA <LOQ	0 0 ND NA	0 0 ND NA	100 0 NA <LOQ	100 A 100 0.690 ± 0.62 0.65–0.73	100^B^ 100 0.597 ± 0.02 0.58–0.61
**Chinook Salmon**	4	% Detect % > LOQ Mean±SD Min-max	0 0 ND NA	0 0 ND NA	0 0 ND NA	0 0 ND NA	0 0 ND NA	50 0 NA <LOQ	25 0 NA <LOQ	0 0 ND NA	100 0 NA <LOQ	100 0 NA <LOQ
**Chum Salmon**	5	% Detect % > LOQ Mean±SD Min-max	0 0 ND NA	0 0 ND NA	0 0 ND NA	20 0 NA <LOQ	0 0 ND NA	20 0 NA <LOQ	20 0 NA <LOQ	0 0 ND NA	0 0 ND NA	0 0 ND NA
**Coho Salmon**	5	% Detect % > LOQ Mean±SD Min-max	0 0 ND NA	0 0 ND NA	0 0 ND NA	0 0 ND NA	0 0 ND NA	0 0 ND NA	0 0 ND NA	0 0 ND NA	0 0 ND NA	0 0 ND NA
**Pacific Cod**	5	% Detect % > LOQ Mean±SD Min-max	0 0 ND NA	0 0 ND NA	0 0 ND NA	0 0 ND NA	20 0 NA <LOQ	0 0 ND NA	0 0 ND NA	0 0 ND NA	100 0 NA <LOQ	100 0 NA <LOQ
**Pacific Halibut**	5	% Detect % > LOQ Mean±SD Min-max	0 0 ND NA	0 0 ND NA	0 0 ND NA	0 0 ND NA	0 0 ND NA	0 0 ND NA	20 0 NA <LOQ	0 0 ND NA	0 A 0 ND NA	100^B^ 0 NA <LOQ
**Pink Salmon**	5	% Detect % > LOQ Mean±SD Min-max	0 0 ND NA	0 0 ND NA	0 0 ND NA	0 0 ND NA	0 0 ND NA	0 0 ND NA	40 0 NA <LOQ	0 0 ND NA	0 0 ND NA	20 0 NA <LOQ
**Pollock**	7	% Detect % > LOQ Mean±SD Min-max	0 0 ND NA	0 0 ND NA	0 0 ND NA	14.3 0 NA <LOQ	0 0 ND NA	28.6 0 NA <LOQ	57.4 C 14.3* 0.209 ± 0.27 <LOQ−0.81	0 0 ND NA	14.3 0 NA <LOQ	100 0 NA <LOQ
**Red King Crab**	1	% Detect % > LOQ Mean±SD Min-max	0 0 ND NA	0 0 ND NA	0 0 ND NA	0 0 ND NA	0 0 ND NA	0 0 ND NA	0 0 ND NA	0 0 ND NA	0 0 ND NA	0 0 ND NA
**Sablefish**	7	% Detect % > LOQ Mean±SD Min-max	14.3^E^ 0 NA <LOQ	0 0 ND NA	0 0 ND NA	0 0 ND NA	0 0 ND NA	28.6^D^ 0 NA <LOQ	0 0 ND NA	0 0 ND NA	57.1 A 0 NA <LOQ	0 0 ND NA
**Sockeye Salmon**	5	% Detect % > LOQ Mean±SD Min-max	0 0 ND NA	0 0 ND NA	0 0 ND NA	0 0 ND NA	0 0 ND NA	20 0 NA <LOQ	20 0 NA <LOQ	0 0 ND NA	100 0 NA <LOQ	100 0 NA <LOQ
**All Species**	51	% Detect % > LOQ Mean±SD Min-max	2 0 NA <LOQ	4 0 NA <LOQ	2 0 NA <LOQ	4 0 NA <LOQ	5.9 0 NA <LOQ	15.7 0 NA <LOQ	19.6 2.0 NA <LOQ	4 0 NA <LOQ	41.2 3.9 NA <LOQ	56.9 3.9 NA <LOQ
		Avg. LOQ (ng/g)	2.9	0.39	0.39	0.405	0.39	0.39	0.39	0.39	0.393	0.393

EPA Method 1633 A and SGS AXYS method MLA−110

Composite samples contained equal portions of 12 individuals

Means were calculated using ½ MDL to estimate NDs.

LOQ = Limit of Quantification

MDL = Method Detection Limit

Compounds not detected: PFBA, PFPeA, PFHxA, PFHpA, PFTeDA, PFBS, PFPeS, PFHpS, PFNS, PFDS, PFDoS, 4:2 FTS, 6:2 FTS, 8:2 FTS, N-MeFOSA, N-EtFOSA, MeFOSAA, EtFOSAA, N-MeFOSE, HFPO-DA, ADONA, 9Cl-PF3ONS, and 11Cl-PF3OUdS

A Laboratory procedural blank = 0.152 ng/g

B Laboratory procedural blank = 0.167 ng/g

C Laboratory procedural blank = 0.154 ng/g

D Laboratory procedural blank = 0.121 ng/g

E 1 out of 7 samples, maximum possible value (“R” laboratory qualifier flag)

* One composite sample out of 7 was above the LOQ (0.809 ng/g), duplicate sample result for this composite was 0.213 ng/g

### 3.1 Whitefish

*Pacific halibut*: PFOS was detected in one composite sample and PFUnA was detected in all five composites of Pacific halibut. Concentrations were below the LOQ and the mean values resulted in non-detections for all PFAS (Table 3). *Pacific Cod*: PFNA was detected in one of five composites of Pacific cod, below the LOQ. Both PFTrDA and PFUnA were detected in all composites below the LOQ. Laboratory blank values were 0.152 and 0.167 ng/g for PFTrDA and PFUnA, respectively. *Sablefish*: One out of seven sablefish composites had detectable N-EtFOSE although the result was flagged as not meeting qualification criteria and is considered a maximum possible concentration. Two composites had detectable PFOA and four composites had PFTrDA, all below the LOQ. Blank values were: PFOA, 0.121 ng/g and PFTrDA, 0.152 ng/g. *Pollock*: The five PFAS detected in pollock were PFHxS, PFOA, PFOS, PFTrDA, and PFUnA. PFHxS was detected above the MDL in one of the seven composites but below the LOQ. PFOA was detected in two composites, but with concentrations below the LOQ. Of the four composites with PFOS above the MDL, only one was above the LOQ at 0.809 ng/g. This sample had a duplicate and the concentration was 0.213 ng/g, less than the LOQ and resulting in a relative percent difference (RPD) greater than 40%. The mean concentration was 0.511 ng/g. The measured values were near the MDL and incomplete homogenization is a likely factor contributing to the large difference in duplicates. PFOS was also detected in the lab blank at a concentration of 0.154 ng/g. PFTrDA was only detected in one composite, while PFUnA was found in all pollock composites, but below the LOQ. PFUnA was found in the lab blank at a concentration of 0.143 ng/g.

### 3.2 Salmon

Coho salmon had no PFAS above the MDL. Two of five pink salmon composites had PFOS greater than the MDL and one composite had PFUnA, but all below the LOQ. PFHxS, PFOA, and PFOS were detected in one of five chum salmon composites, but all were less than the LOQ. PFOA and PFOS were found in one of five sockeye salmon composites, also below the LOQ. All sockeye salmon and chinook salmon composites had PFTrDA and PFUnA above the MDL, but less than the LOQ. Chinook salmon also had two of four composites with PFOA and one with PFOS that were just above the MDLs and contaminated blanks for both compounds (Table 3).

### 3.3 Crab

*Red King Crab*: No levels of PFAS above the MDL were found in red king crab. *Bairdi Crab*: One of two composite samples had PFDoA detected. Both composites had PFDA, PFNA, PFOSA, PFTrDA, and PFUnA. PFTrDA and PFUnA were above the LOQ in all composites (Table 3). Laboratory blank concentrations were 0.152 and 0.167 ng/g for PFTrDA and PFUnA, respectively.

## 4 Discussion

This study provides preliminary baseline PFAS concentrations in edible tissue of fish species harvested from Alaska’s commercial fisheries that are utilized in markets worldwide. Because these samples came directly from seafood supply lines and were typically obtained before secondary processing (cooking, canning, packaging, etc.), they closely reflect PFAS levels in fish from Alaska’s marine environment. The study results indicate that PFAS levels in Alaska’s marine fish and shellfish are low and not likely a significant source of dietary exposure. Our findings are consistent with previously unpublished data collected by the SOA (see Appendix A, Table 3) and other Alaska focused studies [27].

### 4.1 Specific compounds

PFTrDA was the most commonly detected compound (57% of samples), although mostly below the LOQ. PFUnA was the next most common in 41% of samples. PFTrDA and PFUnA are long-chain perfluoroalkyl carboxylic acids with 13 and 11 carbon atoms (respectively), known to bioaccumulate [13], [29], [41] and are commonly found in fish [21]. There appears to be a trend for some Arctic fish species, with increasing detections of long-chain PFAS such as PFTrDA and PFUnA, with a concurrent decline in concentrations of PFOS and PFOA [14], [15], [21], [45], [8]. The phase out of PFOS and related compounds has likely contributed to this trend. The same trend is noted in human populations since the phase out, with PFOS serum concentrations steadily decreasing in the last 20 years [59]. The results of this study agree with the general PFOS trend, but all PFAS concentrations were very low.

PFHxS was found in a small percentage of pollock and chum salmon, all below the LOQ. This is in contrast to a recent retail seafood study within the U.S. Bedi et al., [8] in which PFHxS was the most commonly detected PFAS compound. Other market studies did not find PFHxS to be a prominent contaminant [16], [48], [56], [57], [75]. Due to the nature of market studies, the origins of the products tested were variable and largely unknown, indicating that contamination during processing, packaging, or analysis could not be ruled out. PFHxS was targeted for phase out in the U.S. but is still found in some products. Imported products may also be sources of PFHxS [70].

PFOS is one of the most common PFAS compounds found in fish. In the U.S., PFOS has been detected in many wild fish species in San Francisco Bay [46], throughout the continental U.S. states [7], and in canned tuna [48]. In our study, it was detected (>MDL) in less than 20% of all samples and was only above the LOQ in one pollock composite (Table 3). This is consistent with other historical data from Alaskan fish (Supplemental Tables 2 and 3). While PFOS is routinely detected in studies from other parts of the world, detections in Alaska marine fish remain low and are unlikely to be a significant dietary source.

### 4.2 Implications for Alaska’s fishery resources

Our results are similar to market basket studies by U.S. Department of Agriculture (USDA) and others measuring PFAS in seafood samples, i.e., PFAS were not detected, or at very low concentrations in seafood [30], [31], [75], [74]. There were exceptions in these other studies, with some higher PFAS levels, particularly in freshwater species and some imported products. Direct species comparisons of our PFAS data to market basket samples were difficult because of the variable products tested, varying sources and timeframes, common names shared by different species (e.g., “Halibut” for both Pacific and Atlantic halibut), the potential of misidentifying species in the retail market [44], [72], and evolving analytical methods across the PFAS analytical landscape. Additionally, there was a wide range of compounds detected depending on the study, location, analytical methods, and species. While market basket studies are useful to determine what contaminants are present in a sample of the food supply at the consumer level, they do not provide enough information to help consumers make data driven choices when considering what type of seafood to purchase. They also lack the detail and precision to help public health officials derive informed fish consumption recommendations. The sample design of this study addresses some of those issues by targeting collections closer to harvest, prior to secondary processing and covering several commercially significant Alaska seafood species.

Pacific salmon are the most consumed type of seafood in Alaska as well as the most commonly consumed Alaskan seafood in the U.S. as a whole. Additionally, salmon represents a large percentage of seafood exported from the state. In contrast, in the U.S. market, most of the salmon purchased is Atlantic salmon (*Salmo salar*) produced from aquaculture [42]. Consequentially, a majority of PFAS data address farmed Atlantic salmon while data for Pacific salmon is lacking. This study provides a sound baseline for the five species of Pacific salmon. As a fatty fish, salmon generally have low levels of PFAS [56], [75] and this study confirms that all five species of Pacific salmon sampled from across the State of Alaska marine environment are similar, with no PFAS compounds found above the LOQ (Table 3).

Pollock represents the largest fishery in the U.S. by volume [47]. Our results show low levels of PFAS in pollock, with most samples below the MDL. PFOS was the only compound measured above the LOQ and this was only in 1 of 7 composite samples (14%, Table 3). Similar PFOS tissue concentrations were found in other studies with limited market basket pollock samples [75], [8].

### 4.3 Exposure risk

Fish consumption guidelines or advisories are intended to protect fish consumers by restricting fish consumption to a level that presents the least amount of risk to a specific contaminant. Most fish consumption guidelines and advisories focus on risk reduction by establishing a reference dose from a dose-response relationship of a chemical and then incorporating various uncertainty factors. However, this approach ignores the nutritional benefits of consuming fish and instead encourages avoidance of fish, directly or indirectly, to be protective of the most sensitive members of the population [64]. Despite this approach, the selection criteria for primary studies, uncertainty factors, and calculations are not standardized across contaminants or regulatory and health agencies. An additional factor specifically for fish consumption is the unknown bioavailability of PFAS from fish tissue. Uptake from water is used as a proxy which introduces uncertainty to the exposure risk evaluation. As a result, varying approaches are used to determine reference doses applied to fish consumption, complicating how public health agencies determine consumption guidance and communicate them to fish consumers.

Numerous states and government entities around the world have generated consumption recommendations for PFAS, but these numbers span orders of magnitude [36]. For example, the European Union TWI for the sum of PFOS, PFOA, PFNA, and PFHxS from food at 4.4 ng/kg BW/week equates to 352 ng ∑4 PFAS for an 80 kg individual per week. These guidelines also set a maximum concentration for the sum of these four compounds at 2 ng/g wet weight (can vary by species) for fish muscle for consumption by children [23]. In contrast to this, the Food Safety Commission of Japan’s (FSCJ) TDI from food of 20 ng/kg BW/Day for PFOS, and 20 ng/kg BW/day PFOA equates to a TWI of 22,400 ng ∑PFOS and PFOA for an 80 kg individual per week [26]. This equates to a TWI that is almost 64 times more permissive than the EU’s TWI. The variability in guidance presents significant challenges in providing consumption advice and communicating health risks and benefits, depending on which guidelines are referenced.

In this study, the PFAS concentrations for one sample of pollock of 0.809 ng/g were the highest measured, but did not exceed the EU’s maximum allowable level of 2.0 ng/g wet weight for PFOS in fish for consumption by children [23]. Pollock had a mean of 0.401 ng/g for the sum of PFOS, PFOA, PFNA, PFHxS. Applying the U.S. FDA and EPA recommendations for seafood intake of 340 g/week (three servings of 4 oz for pregnant women (https://www.fda.gov/food/consumers/advice-about-eating-fish)) would result in a TWI of 3.44 ng/kg BW/week, putting pollock below the EU’s TWI. Utilizing the FSCJ guidance, pollock wouldn’t even register as a potential risk by any calculation. While children are unlikely to ever consume enough of one species of fish to reach levels of concern for PFAS given the data presented here, this demonstrates the drastic differences of interpretation of the same data using different consumption recommendations.

Although fish can contribute to dietary PFAS exposure, they also provide many essential nutrients and lipids that are critical to health and development, including Vitamins D and B12, selenium, iodine, and Omega−3 fatty acids. Many of the nutrients are unique to fish or are found at elevated levels compared to other foods. These nutritional benefits generally outweigh the risks posed by trace contaminants, a pattern demonstrated in mercury risk-benefit analysis evaluations [62], [64]. The same principle applies to PFAS: low-level contamination does not negate the substantial health benefits of fish consumption [35], [61]. Without more comprehensive PFAS-specific risk-benefit analysis, public health agencies must consider whether advising people to avoid fish because of trace levels of PFAS actually reduces harm, or whether it inadvertently increases health risks by discouraging intake of nutrient-rich foods [61].

Characterizing how much seafood people actually eat is essential when estimating exposure to PFAS from diet. Most Americans eat less than the recommended 8 oz (227 g) of seafood per week [67], making seafood an unlikely source of significant PFAS exposure for the general population in the U.S. Some communities that live a subsistence lifestyle, such as those living near the coast in Alaska, consume significantly more, sometimes exceeding 1000 g/week. Even for these high-consumption cohorts, the data presented here do not indicate a significant exposure risk. Specifically for pollock at Alaska’s high consumption rate (Table 4) was the only species to exceed the EU’s ∑4 PFAS threshold. Pollock is rarely targeted as a subsistence species with salmon and halibut being the most common marine species harvested [51].

**Table 4:** Estimated weekly intake (EWI ng/Kg body weight) of ∑PFAS based on various consumption rates.

**Species**	PFOS + PFOA + PFNA + PFHxS (ng/g w/w)*	**US National**^a^	**US National Seafood consumers**^a^	EU**	**Alaska**^b^
Average consumption per week		**126 g**	**748 g**	**450.7 g**	**1043 g**
C. bairdi crab	0.286	0.45	2.67	1.61	3.73
Chinook Salmon	0.251	0.40	2.35	1.41	3.27
Chum Salmon	0.294	0.46	2.75	1.66	3.83
Coho Salmon	0.196	0.31	1.83	1.10	2.56
Pacific Cod	0.221	0.35	2.07	1.24	2.88
Pacific Halibut	0.249	0.39	2.33	1.40	3.25
Pink Salmon	0.253	0.40	2.36	1.42	3.30
Pollock	0.401	0.63	3.75	2.26	5.23
Red King Crab	0.196	0.31	1.83	1.10	2.56
Sablefish	0.226	0.36	2.11	1.27	2.95
Sockeye Salmon	0.227	0.36	2.12	1.28	3.00

EWI = ((Concentration*Weekly consumption)/Body weight)

Based on adult body weight of 80Kg.

EU TWI of ∑(PFOS, PFOA, PFNA, PFHxS) 4.4 ng/Kg BW [20].

FSCJ Tolerable Daily Intake (TDI) of ∑PFOS and PFOA 40 ng/Kg BW, equivalent to a TWI of 280 ng/Kg BW [26].

* Sum of all four compounds, half the detection limit was used to estimate non-detect results (see Table 3 and Supplementary Table 1).

** [22]

a [43]

b [51]

Accurately identifying the source of the PFAS contamination is critical to risk-benefit analysis and subsequent fish consumption guidelines. PFAS can enter seafood via two distinct pathways: environmental bioaccumulation and contamination during post-harvest handling or processing. While environmental exposure is inherent to the ecosystem from which fish are harvested [60], contamination introduced during processing can potentially be mitigated to minimize contamination. Species and harvest location are both important factors to consider when selecting seafood with lower environmental exposure [52]. PFAS levels can vary widely between freshwater and marine environments [7], [73], proximity to manufacturing and disposal sites [66], and regions affected by long-range transport of PFAS [1]. Due to the variable distribution of PFAS compounds in the environment, source traceability is an important consideration, illustrated by the high PFOA levels in processed clams from China’s Bohai Sea region that led to the FDA denying import for those products to the U.S. [33], [71]. While the data presented here offers a critical baseline assessment of 11 important species of fish from three major marine environments of Alaska, more monitoring data are needed to identify potential hotspot areas, high-risk regions, or uniquely contaminated species. Understanding the source and magnitude of contamination is critical for informing public health guidance and helping consumers make appropriate seafood choices, curb general fears of contaminants in seafood.

### 4.4 Limitations

This study is not without limitations. These include small sample sizes and the use of composite samples. When using composite samples, some precision is sacrificed for other factors, such as logistics or costs. Composite analysis is built on the premise that a sample is generally representative of the population. However, the concentrations of contaminants across fish are inherently variable. Composite samples can be considered representative of potential contaminant exposure, providing an average which incorporates the range of concentrations occurring in fish that consumers eat. Despite losing some data granularity, composites are largely representative and more cost effective. The logistics of working in remote regions of Alaska made sample acquisition and shipping challenging. Using composite samples was necessary due to cost of analysis, but it also enabled the analysis of more fish. This allowed for baseline monitoring over a wider spatial and taxonomic range, providing for better future comparisons. These limitations resulted in small sample sizes, which limited our ability to comprehensively assess fish consumption concerns or conduct an in-depth risk assessment such as a Hazard Quotient or Index. Calculations for these assessments were further limited because of the frequency of non-detects and low concentrations.

## 5 Conclusion

Fish and shellfish harvested in Alaska represent a significant portion of seafood consumed in the U.S. and exported to foreign markets. The presence of PFAS in Alaskan fish in this study were consistent with previous studies of seafood from Alaska and other marine environments. PFAS were not detected, or at nominal concentrations in edible tissues of Alaskan commercial seafood. Given the benefits of eating seafood and the minimal PFAS detected, health risk is low for the species tested in this study. Monitoring contaminant data of wild species destined for human consumption is essential to make informed decisions for consumption recommendations and support consumer choice. This is particularly important because of the complex exposure landscape where PFAS exposure can occur. For seafood, the importance of the type of seafood (species), origin of harvest, and post-harvest handling and processing are critical considerations.

## CRediT authorship contribution statement

**Christoff G. Furin:** Writing – review & editing, Writing – original draft, Resources, Methodology, Investigation, Formal analysis, Data curation, Conceptualization. **Andrew P. Cyr:** Writing – review & editing, Formal analysis. **John Burrows:** Writing – review & editing, Resources, Project administration, Funding acquisition. **Robert F. Gerlach:** Writing – review & editing, Supervision, Project administration, Funding acquisition, Conceptualization. **Sarah Coburn:** Writing – review & editing, Supervision, Project administration.

## Declaration of Competing Interest

The authors declare that they have no known competing financial interests or personal relationships that could have appeared to influence the work reported in this paper.

## Data Availability

Data will be made available on request.

## Footnotes

Appendix A Supplementary data associated with this article can be found in the online version at doi:10.1016/j.toxrep.2026.102302.
