# Supplementary material for: Per- and polyfluoroalkyl substances in commercially important marine species of fish and shellfish from Alaska and implications for human exposure
Source: Toxicol Rep. 2026 Jun 25;17:102302. doi: 10.1016/j.toxrep.2026.102302 (PMC13318540; doi:10.1016/j.toxrep.2026.102302)
Supplement: Supplementary file 1 — Supplementary material [file mmc1.docx]

**Supplemental materials for**:

**Title:** Perfluoroalkyl Substances in Commercially Important Marine Species of Fish and Shellfish from Alaska

**Authors:** Christoff G. Furin^1*^, John Burrows^2^, Andrew P. Cyr^3^, Sarah Coburn^1^, and Robert F. Gerlach^1^

**Affiliations:**

1. Alaska Department of Environmental Conservation, Division of Environmental Health, Office of the State Veterinarian, 5251 Dr. Martin Luther King Jr. Ave., Anchorage, AK 99507, USA
2. Alaska Seafood Marketing Institute, 311 N. Franklin St. Suite 200, Juneau, AK 99801, USA
3. Alaska Department of Health, Division of Public Health, Section of Epidemiology, 3601 C Street Suite 540, Anchorage, AK 99503, USA

*****Corresponding Author:

Christoff G. Furin

Email: [christoff.furin@alaska.gov](mailto:christoff.furin@alaska.gov)

**Data availability:** Data is made available publicly.

**Conflicts of interest:** None to declare

**For more information visit:** <https://dec.alaska.gov/eh/vet/fish-monitoring-program/>

**Supplemental Table 1**: PFAS compounds, Method Detection Limits in Fish Tissue and surrogate standards with EPA Method 1633^#^.

| **Abbreviation** | **Name - Acid Form** | **CAS#** | **MDL (ng/g w/w)*** | **Surrogate Standards** |
| --- | --- | --- | --- | --- |
| PFBA | Perfluorobutyric acid | 375-22-4 | 0.39 (0.376, 0.4) | ^13^C_4_-PFBA |
| PFPeA | Perfluoropentanoic acid | 2706-90-3 | 0.195 (0.188, 0.2) | ^13^C_5_-PFPeA |
| PFHxA | Perfluorohexanoic acid | 307-24-4 | 0.097 (0.094, 0.1) | ^13^C5-PFHxA |
| PFHpA | Perfluoroheptanoic acid | 375-85-9 | 0.097 (0.094, 0.1) | ^13^C_4_-PFHpA |
| PFOA | Perfluorooctanoic acid | 335-67-1 | 0.097 (0.094, 0.1) | ^13^C_8_-PFOA |
| PFNA | Perfluorononanoic acid | 375-95-1 | 0.097 (0.094, 0.1) | ^13^C_9_-PFNA |
| PFDA | Perfluorodecanoic acid | 335-76-2 | 0.097 (0.094, 0.1) | ^13^C_6_-PFDA |
| PFUnA | Perfluoroundecanoic acid | 2058-94-8 | 0.097 (0.094, 0.1) | ^13^C_7_-PFUnA |
| PFDoA | Perfluorododecanoic acid | 307-55-1 | 0.078 (0.075, 0.08) | ^13^C_2_-PFDoA |
| PFTrDA | Perfluorotridecanoic acid | 72629-94-8 | 0.097 (0.094, 0.1) |  |
| PFTeDA | Perfluorotetradecanoic acid | 376-06-7 | 0.097 (0.094, 0.1) | ^13^C_2_-PFTeDA |
| PFBS | Perfluorobutanesulfonic acid | 375-73-5 | 0.097 (0.094, 0.1) | ^13^C_3_-PFBS |
| PFPeS | Perfluoropentanesulfonic acid | 2706-91-4 | 0.098 (0.094, 0.1) |  |
| PFHxS | Perfluorohexanesulfonic acid | 355-46-4 | 0.101 (0.094, 0.115) | ^13^C_3_-PFHxS |
| PFHpS | Perfluoroheptanesulfonic acid | 375-92-8 | 0.097 (0.094, 0.1) |  |
| PFOS | Perfluorooctanesulfonic acid | 1763-23-1 | 0.097 (0.094, 0.1) | ^13^C_8_-PFOS |
| PFNS | Perfluorononanesulfonic acid | 68259-12-1 | 0.097 (0.094, 0.1) |  |
| PFDS | Perfluorodecanesulfonic acid | 335-77-3 | 0.097 (0.094, 0.1) |  |
| PFDoS | Perfluorododecanesulfonic acid | 79780-39-5 | 0.097 (0.094, 0.1) |  |
| 4:2 FTS | 4:2 fluorotelomersulfonic acid | 757124-72-4 | 0.39 (0.376, 0.4) | ^13^C_2_-4:2 FTS |
| 6:2 FTS | 6:2 fluorotelomersulfonic acid | 27619-97-2 | 0.35 (0.338, 0.36) | ^13^C_2_-6:2 FTS |
| 8:2 FTS | 8:2 fluorotelomersulfonic acid | 39108-34-4 | 0.33 (0.319, 0.34) | ^13^C_2_-8:2 FTS |
| 3:3 FTCA | 3:3 perfluorohexanoic acid | 356-02-5 | 0.39 (0.376, 0.4) |  |
| 5:3 FTCA | 5:3 perfluorooctanoic acid | 914637-49-3 | 2.44 (2.35, 2.5) |  |
| 7:3 FTCA | 7:3 perfluorodecanoic acid | 812-70-4 | 2.44 (2.35, 2.5) |  |
| NMeFOSAA | N-Methylperfluorooctanesulfonamidoacetic acid | 2355-31-9 | 0.097 (0.0939, 0.1) | D_3_-NMeFOSAA |
| NEtFOSAA | N-Ethylperfluorooctanesulfonamidoacetic acid | 2991-50-6 | 0.097 (0.0939, 0.1) | D_5_-NEtFOSAA |
| HFPO-DA | 2,3,3,3-Tetrafluoro-2-(1,1,2,2,3,3,3-heptafluoropropoxy)propanoic acid | 13252-13-6 | 0.39 (0.376, 0.4) | ^13^C_3_-HFPO-DA |
| ADONA | Dodecafluoro-3H-4,8-dioxanonanoic acid | 919005-14-4 | 0.39 (0.376, 0.4) |  |
| 9Cl-PF3ONS | 9-chlorohexadecafluoro-3-oxanonane-1-sulfonicacid | 756426-58-1 | 0.39 (0.377, 0.401) |  |
| 11Cl-PF3OUdS | 11-chloroeicosafluoro-3-oxaundecane-1-sulfonicacid | 763051-92-9 | 0.39 (0.376, 0.401) |  |
| NFDHA | Perfluoro-3,6-dioxaheptanoic acid | 151772-58-6 | 0.19 (0.188, 0.2) |  |
| PFMPA | Perfluoro-3-methoxypropanoic acid | 377-73-1 | 0.195 (0.188, 0.2) |  |
| PFMBA | Perfluoro-4-methoxybutanoic acid | 863090-89-5 | 0.097 (0.094, 0.1) |  |
| PFEESA | Perfluoro(2-ethoxyethane)sulfonic acid | 113507-82-7 | 0.097 (0.094, 0.1) |  |
| PFOSA | Perfluorooctanesulfonamide | 754-91-6 | 0.097 (0.094, 0.1) | ^13^C_8_-PFOSA |
| NMeFOSA | N-Methylperfluorooctanesulfonamide | 31506-32-8 | 0.10 (0.095, 0.135) | D_3_-NMeFOSA |
| NEtFOSA | N-Ethylperfluorooctanesulfonamide | 4151-50-2 | 0.27 (0.263, 0.28) | D_5_-NEtFOSA |
| NMeFOSE | N-Methylperfluorooctanesulfonamidoethanol | 24448-09-7 | 0.97 (0.939, 1.0) | D_7_-NMeFOSE |
| NEtFOSE | N-Ethylperfluorooctanesulfonamidoethanol | 1691-99-2 | 0.97 (0.939, 1.0) | D_9_-NEtFOSE |

*Mean (min, max) of sample specific detection limits

^#^SGS AXYS Method MLA-110 REV. 02 VER. 16

**Supplemental Table 2**: Percent Detected and mean (ng/g wet weight) PFAS concentrations in fish fillet tissue of fish collected in Alaska marine waters and tested by the State of Alaska prior to 2012.

|  |  |  | **Chinook Salmon** | **Coho Salmon** | **Pacific Cod** | **Pacific Halibut** | **Pacific Herring** | **Eulachon** | **Sockeye Salmon** |
| --- | --- | --- | --- | --- | --- | --- | --- | --- | --- |
| **Analyte** | **RL** | **Statistic** | *Oncorhynchus tshawytscha* | *Oncorhynchus keta* | *Gadus macrocephalus* | *Hippoglossus stenolepis* | *Clupea pallasii* | *Thaleichthys pacificus* | *Oncorhynchus nerka* |
|  | **ng/g** | **n** | **33** | **53** | **5** | **20** | **10** | **7** | **13** |
| PFBA | 2.45 | % Detect  Mean | 0  ND | 0  ND | 0  ND | 0  ND | 0  ND | 0  ND | 0  ND |
| PFPeA | 2.45 | % Detect  Mean | 0  ND | 0  ND | 0  ND | 0  ND | 0  ND | 0  ND | 0  ND |
| PFHxA | 2.45 | % Detect  Mean | 0  ND | 0  ND | 0  ND | 0  ND | 0  ND | 0  ND | 0  ND |
| PFHpA | 2.45 | % Detect  Mean | 0  ND | 0  ND | 0  ND | 0  ND | 0  ND | 0  ND | 0  ND |
| PFOA | 2.45 | % Detect  Mean | 0  ND | 1.9  ND | 0  ND | 0  ND | 0  ND | 0  ND | 0  ND |
| PFNA | 2.45 | % Detect  Mean | 0  ND | 0  ND | 0  ND | 0  ND | 0  ND | 0  ND | 0  ND |
| PFDA | 2.45 | % Detect  Mean | 0  ND | 1.9  ND | 0  ND | 0  ND | 0  ND | 0  ND | 0  ND |
| PFUnA | 2.45 | % Detect  Mean | 0  ND | 0  ND | 0  ND | 0  ND | 0  ND | 0  ND | 0  ND |
| PFDoA | 2.45 | % Detect  Mean | 0  ND | 1.9  ND | 0  ND | 0  ND | 0  ND | 0  ND | 0  ND |
| PFBS | 4.9 | % Detect  Mean | 0  ND | 0  ND | 0  ND | 0  ND | 0  ND | 0  ND | 0  ND |
| PFHxS | 4.9 | % Detect  Mean | 0  ND | 0  ND | 0  ND | 0  ND | 0  ND | 0  ND | 0  ND |
| PFOS | 4.9 | % Detect  Mean | 3.0  ND | 1.9  ND | 0  ND | 0  ND | 0  ND | 0  ND | 0  ND |
| PFOSA | 2.45 | % Detect  Mean | 0  ND | 0  ND | 0  ND | 0  ND | 0  ND | 0  ND | 0  ND |

Fish samples collected before 2012, 13 PFAS compounds tested and Detection Limits are higher than current project, using AXYS method MLA-043 Rev 08 by LC-MS/MS

ng/g wet weight

RL= Reporting Limit

ND = < RL

**Supplemental Table 3**: Percent detection, percent above LOQ, and mean values of PFAS compounds (ng/g wet weight) in composite muscle samples of fish caught Alaska marine waters and tested by the State of Alaska from the years 2012 through 2022.

| Sample | **n** | Statistic | N-  EtFOSE | PFDA | PFDoA | PFHxA | PFNA | PFOA | PFOS | PFOSA | PFTeDA | PFTrDA | PFUnA | 6:2 FTS |
| --- | --- | --- | --- | --- | --- | --- | --- | --- | --- | --- | --- | --- | --- | --- |
| **Alaska Plaice** *Pleuronectes quadrituberculatus* | 5 | % Detect  % > LOQ  Mean±SD  Min-max | 0  0  ND  NA | 0  0  ND  NA | 0  0  ND  NA | 0  0  ND  NA | 100  40  0.36±0.63  <LOQ-0.43 | 0  0  ND  NA | 60  40  0.46±0.53  <LOQ-1.1 | 0  0  ND  NA | 0  0  ND  NA | 0  0  ND  NA | 0  0  ND  NA | 0  0  ND  NA |
| **Atka Mackerel** *Pleurogrammus monopterygius* | 2^C^ | % Detect  % > LOQ  Mean±SD  Min-max | 0  0  ND  NA | 0  0  ND  NA | 0  0  ND  NA | 0  0  ND  NA | 0  0  ND  NA | 0  0  ND  NA | 100  0  NA  <LOQ | 0  0  ND  NA | 0  0  ND  NA | 100  0  NA  <LOQ | 100  0  NA  <LOQ | 0  0  ND  NA |
| **Arrowtooth Flounder** *Atheresthes stomias* | 2^C^ | % Detect  % > LOQ  Mean±SD  Min-max | 0  0  ND  NA | 0  0  ND  NA | 0  0  ND  NA | 0  0  ND  NA | 0  0  ND  NA | 0  0  ND  NA | 100  0  NA  <LOQ | 0  0  ND  NA | 0  0  ND  NA | 0  0  ND  NA | 100  0  NA  <LOQ | 0  0  ND  NA |
| **C. bairdi Crab** *Chionoecetes bairdi* | 1^C^ | % Detect  % > LOQ  Mean±SD  Min-max | 0  0  ND  NA | 100  0  NA  <LOQ | 100  0  NA  <LOQ | 0  0  ND  NA | 100  0  NA  <LOQ | 0  0  ND  NA | 0  0  ND  NA | 100  0  NA  <LOQ | 0  0  ND  NA | 100  0  NA  <LOQ | 100  0  NA  <LOQ | 0  0  ND  NA |
| **Chinook Salmon** *Oncorhynchus tshawytscha* | 1^C^ | % Detect  % > LOQ  Mean±SD  Min-max | 0  0  ND  NA | 0  0  ND  NA | 0  0  ND  NA | 0  0  ND  NA | 0  0  ND  NA | 0  0  ND  NA | 100  0  NA  <LOQ | 0  0  ND  NA | 0  0  ND  NA | 100  0  NA  <LOQ | 100  0  NA  <LOQ | 0  0  ND  NA |
| **Chum Salmon** *Oncorhynchus keta* | 1^C^ | % Detect  % > LOQ  Mean±SD  Min-max | 0  0  ND  NA | 0  0  ND  NA | 0  0  ND  NA | 0  0  ND  NA | 0  0  ND  NA | 0  0  ND  NA | 0  0  ND  NA | 0  0  ND  NA | 0  0  ND  NA | 0  0  ND  NA | 0  0  ND  NA | 0  0  ND  NA |
| **Flathead Sole** *Hippoglossoides elassodon* | 5 | % Detect  % > LOQ  Mean±SD  Min-max | 80  0  NA  <LOQ | 40  0  NA  <LOQ | 0  0  ND  NA | 0  0  ND  NA | 100  40  0.86±0.56  <LOQ-1.63 | 80  0  NA  <LOQ | 60  0  NA  <LOQ | 0  0  ND  NA | 0  0  ND  NA | 0  0  ND  NA | 40  0  NA  <LOQ | 0  0  ND  NA |
| **Northern Rock Sole** *Lepidopsetta polyxystra* | 5 | % Detect  % > LOQ  Mean±SD  Min-max | 20  0  NA  <LOQ | 0  0  ND  NA | 0  0  ND  NA | 0  0  ND  NA | 40  20  0.14±0.18  <LOQ-0.47 | 0  0  ND  NA | 80  0  NA  <LOQ | 0  0  ND  NA | 0  0  ND  NA | 0  0  ND  NA | 0  0  ND  NA | 0  0  ND  NA |
| **Pacific Halibut** *Hippoglossus stenolepis* | 3^C^ | % Detect  % > LOQ  Mean±SD  Min-max | 0  0  ND  NA | 0  0  ND  NA | 0  0  ND  NA | 0  0  ND  NA | 33.3  0  NA  <LOQ | 0  0  ND  NA | 0  0  ND  NA | 0  0  ND  NA | 0  0  ND  NA | 66.6  0  NA  <LOQ | 66.6  0  NA  <LOQ | 0  0  ND  NA |
| **Pacific Herring** *Clupea pallasii* | 1^C^ | % Detect  % > LOQ  Mean±SD  Min-max | 100  0  NA  <LOQ | 0  0  ND  NA | 0  0  ND  NA | 100  0  NA  <LOQ | 0  0  ND  NA | 0  0  ND  NA | 0  0  ND  NA | 0  0  ND  NA | 0  0  ND  NA | 0  0  ND  NA | 0  0  ND  NA | 100  0  NA  <LOQ |
| **Pacific Ocean Perch**  *Sebastes alutus* | 2^C^ | % Detect  % > LOQ  Mean±SD  Min-max | 0  0  ND  NA | 0  0  ND  NA | 0  0  ND  NA | 0  0  ND  NA | 0  0  ND  NA | 0  0  ND  NA | 50  0  ND  <LOQ | 0  0  ND  NA | 0  0  ND  NA | 100  0  NA  <LOQ | 100  0  NA  <LOQ | 0  0  ND  NA |
| **Pacific Octopus** *Enteroctopus dofleini* | 2^C^ | % Detect  % > LOQ  Mean±SD  Min-max | 0  0  ND  NA | 0  0  ND  NA | 100  0  ND  <LOQ | 0  0  ND  NA | 0  0  ND  NA | 0  0  ND  NA | 0  0  ND  NA | 0  0  ND  NA | 0  0  ND  NA | 100  100  0.64±0.02  0.62-0.65 | 100  50  0.37±0.10  <LOQ-0.44 | 0  0  ND  NA |
| **Pollock**  *Gadus chalcogrammus* | 2^C^ | % Detect  % > LOQ  Mean±SD  Min-max | 0  0  ND  NA | 0  0  ND  NA | 0  0  ND  NA | 0  0  ND  NA | 0  0  ND  NA | 0  0  ND  NA | 0  0  ND  NA | 0  0  ND  NA | 0  0  ND  NA | 50  0  NA  <LOQ | 100  0  NA  <LOQ | 0  0  ND  NA |
| **Sablefish** *Anoplopoma fimbria* | 4^C^ | % Detect  % > LOQ  Mean±SD  Min-max | 0  0  ND  NA | 0  0  ND  NA | 0  0  ND  NA | 0  0  ND  NA | 0  0  ND  NA | 0  0  ND  NA | 25  0  ND  <LOQ | 0  0  ND  NA | 25  0  ND  <LOQ | 50  0  NA  <LOQ | 0  0  ND  NA | 0  0  ND  NA |
| **Sockeye Salmon** *Oncorhynchus nerka* | 1^C^ | % Detect  % > LOQ  Mean±SD  Min-max | 0  0  ND  NA | 0  0  ND  NA | 0  0  ND  NA | 0  0  ND  NA | 0  0  ND  NA | 0  0  ND  NA | 0  0  ND  NA | 0  0  ND  NA | 0  0  ND  NA | 0  0  ND  NA | 0  0  ND  NA | 0  0  ND  NA |
| **Squid spp.** *Unknown spp.* | 2^C^ | % Detect  % > LOQ  Mean±SD  Min-max | 0  0  ND  NA | 0  0  ND  NA | 0  0  ND  NA | 0  0  ND  NA | 0  0  ND  NA | 0  0  ND  NA | 0  0  ND  NA | 0  0  ND  NA | 0  0  ND  NA | 0  0  ND  NA | 0  0  ND  NA | 0  0  ND  NA |
| **Yellowfin Sole** *Limanda aspera* | 5 | % Detect  % > LOQ  Mean±SD  Min-max | 0  0  ND  NA | 60  0  NA  <LOQ | 0  0  ND  NA | 0  0  ND  NA | 100  20  0.34±0.05  <LOQ-0.39 | 20  0  NA  <LOQ | 100  60  0.43±0.11  <LOQ-0.57 | 0  0  ND  NA | 0  0  ND  NA | 20  0  NA  <LOQ | 80  0  NA  <LOQ | 0  0  ND  NA |
|  |  | LOQ (ng/g) | 3.24 | 0.41 | 0.40 | 0.41 | 0.41 | 0.41 | 0.41 | 0.41 | 0.41 | 0.41 | 0.41 | 3.90 |

Analytical method equivalent to current study (EPA Method 1633A and SGS AXYS method MLA-110).

Means were calculated using ½ MDL to estimate NDs.

LOQ = Limit of Quantification

ND = Non-detect

NA = Not applicable

^C^ = Composite sample

Compounds not detected: PFBA, PFPeA, PFHpA, PFBS, PFPeS, PFHxS, PFHpS, PFNS, PFDS, PFDoS, 4:2 FTS, 8:2 FTS, N-MeFOSA, N-EtFOSA, MeFOSAA, EtFOSAA, N-MeFOSE, HFPO-DA, ADONA, 9Cl-PF3ONS, and 11Cl-PF3OUdS.


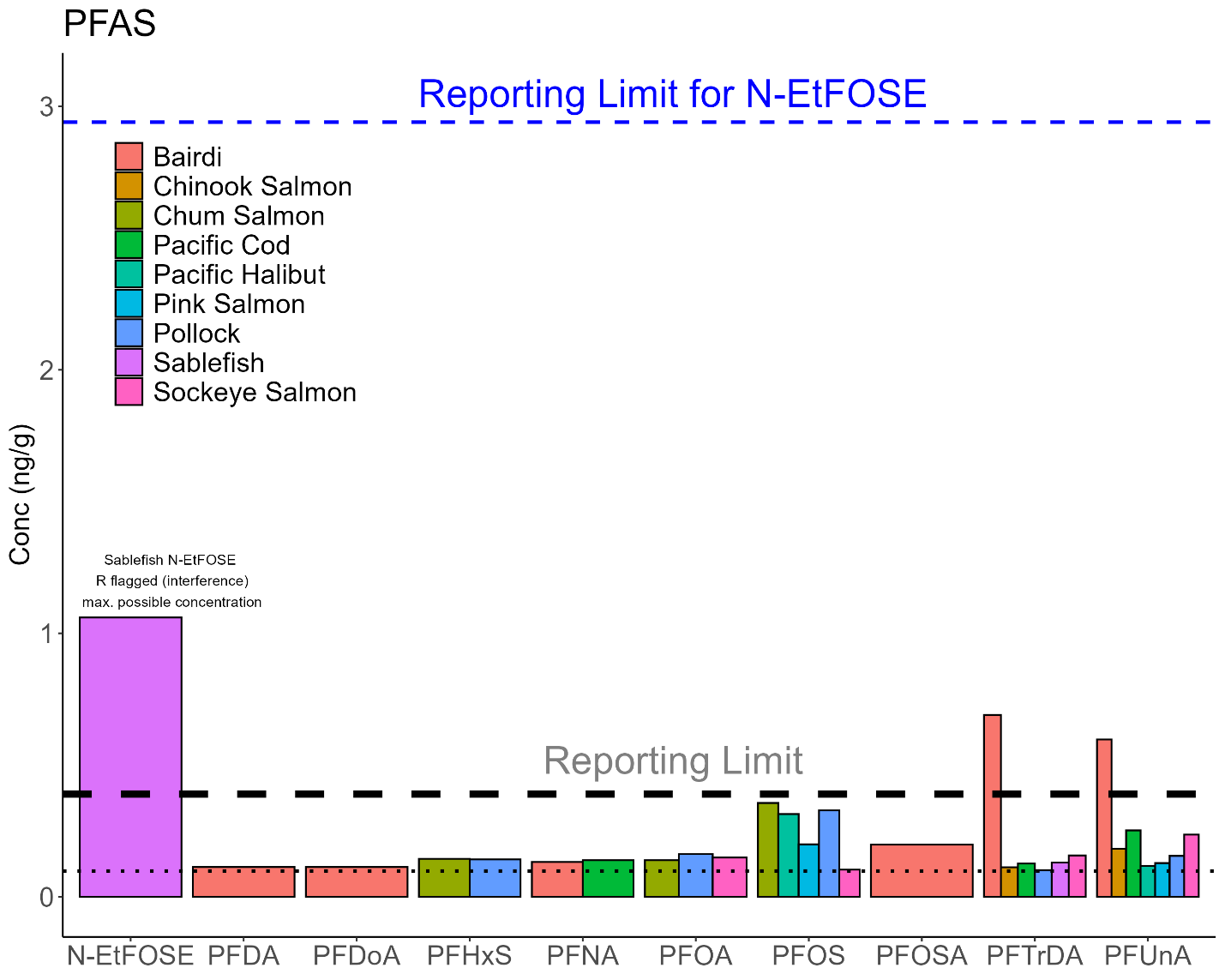


**Supplemental Figure 1:** Mean (ng/g wet weight) concentration of PFAS compounds detected in fish and shellfish from Alaska marine waters. Dotted line is the mean MDL.


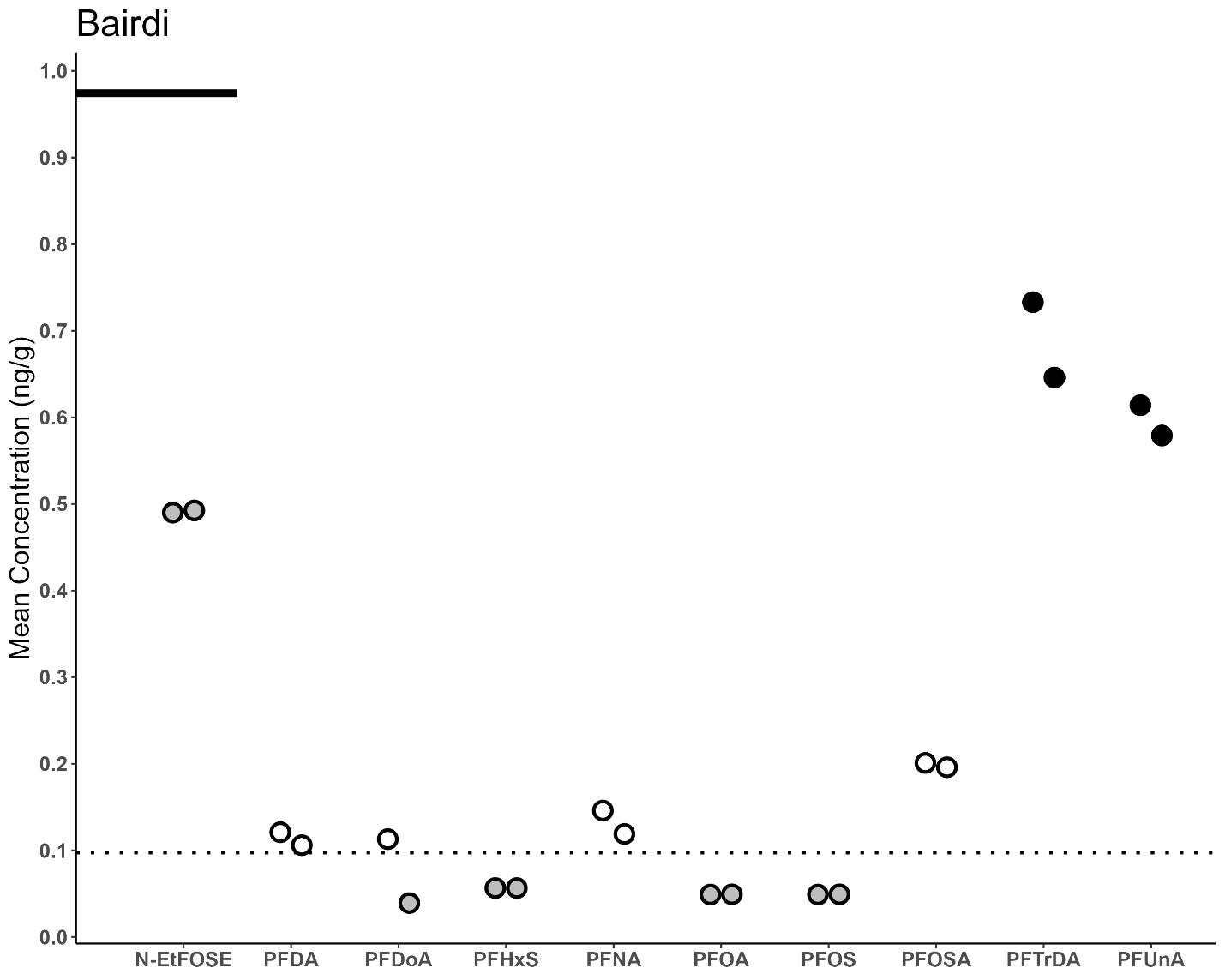


**Supplemental Figure 2:** PFAS concentrations is muscle of *C. bairdi*. Solid black line is the MDL for N-EtFOSE, dotted line is the mean MDL for all other compounds. Gray circles are below the MDL (= ½ MDL), white circles are below the LOQ and black circles are above the LOQ. See Supp. Table 1 and Table 3 for MDL and LOQ values. Laboratory procedural blank concentrations: PFUnA-0.167 ng/g; PFTrDA-0.152 ng/g


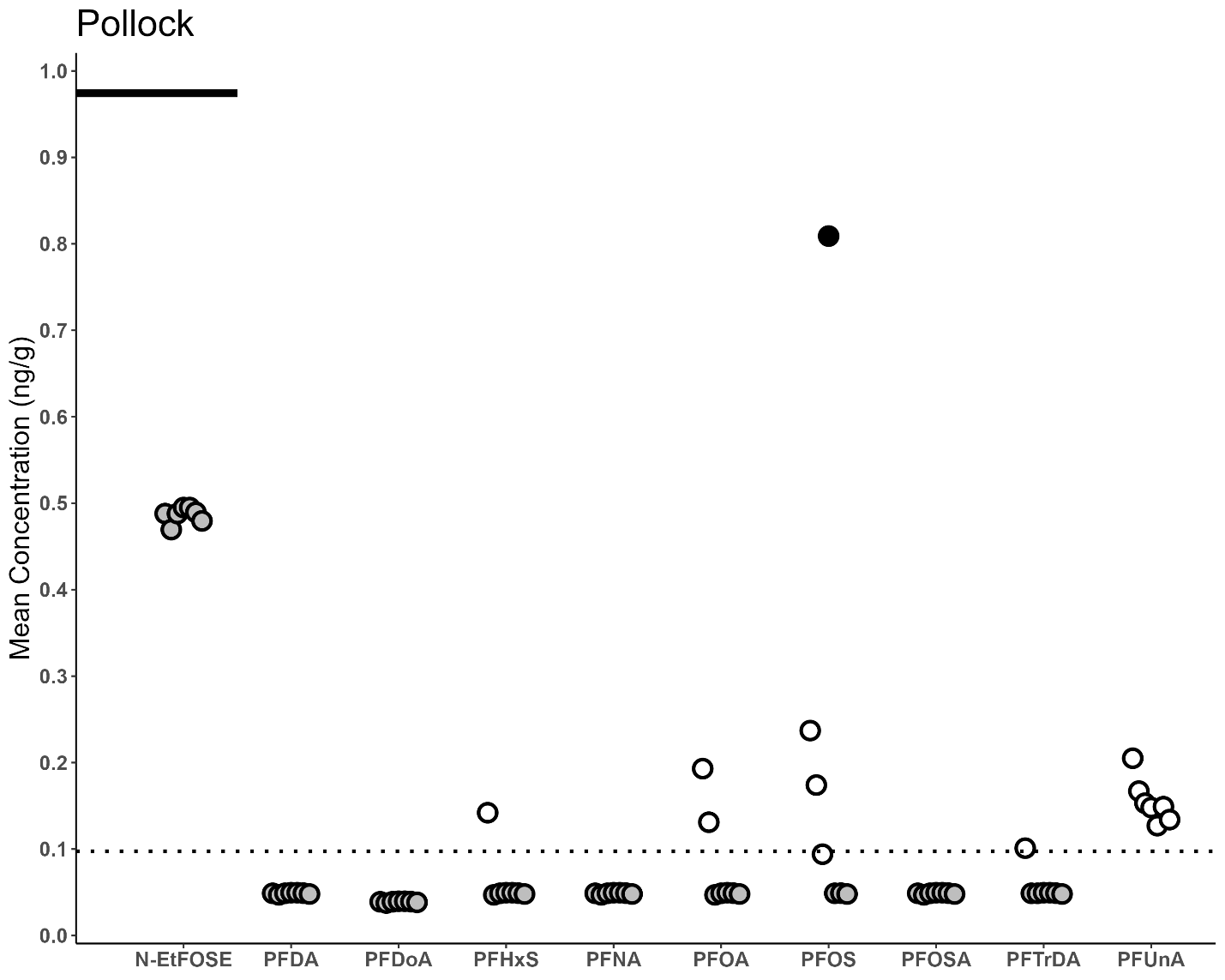


**Supplemental Figure 3:** PFAS concentrations in muscle of Alaska Pollock. Solid black line is the MDL for N-EtFOSE, dotted line is the mean MDL for all other compounds. Gray circles are below the MDL = (½ MDL), white circles are below the LOQ and black circles are above the LOQ. See Supp. Table 1 and Table 3 for MDL and LOQ values. Laboratory procedural blank concentrations: PFOS-0.154 ng/g; PFUnA-0.137 ng/g
